# Bacteria in a wood fungal disease: characterization of bacterial communities in wood tissues of esca-foliar symptomatic and asymptomatic grapevines

**DOI:** 10.3389/fmicb.2015.01137

**Published:** 2015-10-27

**Authors:** Emilie Bruez, Rana Haidar, Maryam T. Alou, Jessica Vallance, Christophe Bertsch, Flore Mazet, Marc Fermaud, Alain Deschamps, Lucia Guerin-Dubrana, Stéphane Compant, Patrice Rey

**Affiliations:** ^1^Institut des Sciences de la Vigne et du Vin, Université de BordeauxBordeaux, France; ^2^UMR1065 Santé et Agroécologie du Vignoble, Bordeaux Sciences AgroGradignan, France; ^3^Institut National de la Recherche Agronomique, Institut des Sciences de la Vigne et du Vin, UMR1065 Santé et Agroécologie du VignobleVillenave d’Ornon, France; ^4^Laboratoire Vigne Biotechnologie et Environnement EA-3991, Université de Haute-AlsaceColmar, France; ^5^Bioresources Unit, Health and Environment Department, AIT Austrian Institute of Technology GmbHTulln, Austria

**Keywords:** bacterial communities, fungal disease, esca, microbiology, grapevine

## Abstract

Esca is a grapevine trunk disease (GTD) associated with different pathogenic fungi inhabiting the woody tissues. Bacteria can also be found in such tissues and they may interact with these fungal colonizers. Although such types of microbial interactions have been observed for wood diseases in many trees, this has never been studied for grapevine. In this study, the bacterial microflora of different vine status (esca-symptomatic and asymptomatic), different anatomical part (trunk and cordon) and different type of tissues (necrotic or not) have been studied. Based on Single Strand Conformation Polymorphism (SSCP) analyses, data showed that (i) specific complexes of bacterial microflora colonize the wood of both necrotic and non-necrotic tissues of esca-foliar symptomatic and asymptomatic vines, and also that (ii) depending on the anatomical part of the plant, cordon or trunk, differences could be observed between the bacterial communities. Such differences were also revealed through the community-level physiological profiling (CLPP) with Biolog Ecoplates^TM^. Two hundred seventeen bacterial strains were also isolated from plant samples and then assigned to bacterial species based on the 16S rRNA genes. Although *Bacillus* sp. and *Pantoea agglomerans* were the two most commonly isolated species from all kinds of tissues, various other taxa were also isolated. Inoculation of vine cuttings with 14 different bacterial species, and one GTD fungus, *Neofusicoccum parvum*, showed no impact of these bacteria on the size of the wood necroses caused by *N. parvum.* This study showed, therefore, that bacterial communities differ according to the anatomical part (trunk or cordon) and/or the type of tissue (necrotic or non-necrotic) of wood of grapevine plants showing external symptoms of esca disease. However, research into bacteria having a role in GTD development needs further studies.

## Introduction

Viticulture is now confronted with major challenges in the form of global climate change, grapevine trunk diseases (GTDs: esca, Eutypa and Botryosphaeria dieback), and strong consumer demand for environmentally friendly viticulture. GTDs and particularly esca have been known since antiquity but, over the last two decades, they have become a subject of major concern for the wine industry. Esca attacks the perennial organs of the plants, producing extensive wood necroses in the trunk and cordon. Typical symptoms can be observed on leaves (“tiger-stripe" discolorations) as well as on berries (small size, black measles, sugar content, and affect flavor). Grapevine plant growth may decline and then die. According to Hofstetter et al. (2012), the cost of replacing plants affected by GTDs exceeds, worldwide, 1.132 billion euros per year. Esca has been associated with fungal species such as *Phaeomoniella chlamydospora*, *Phaeoacremonium aleophilum*, *Fomitiporia mediterranea*, *Stereum hirsutum*, and Botryosphaeriaceae species, such as *Neofusicoccum parvum* (Crous et al., 1996; Larignon and Dubos, 1997; Fischer, 2006; Mostert et al., 2006; Pouzoulet et al., 2013). The etiology of the disease, however, still remains poorly understood. It is not even known whether microbes, other than fungi, could interact *in planta* with GTD-associated agents, nor what exactly such types of microorganisms are.

The involvement of microorganisms other than fungi in esca disease is still a matter of speculation. Bacteria have been described as inhabitants of various tissues of all plants studied. Certain bacterial colonizers that induce beneficial effects on their hosts, have an agronomical interest as plant growth promoters (Long et al., 2008) by limiting and/or preventing attacks by phytopathogens (Compant et al., 2005; Hardoim et al., 2015). Others, however, may have neutral or pathogenic effects (Hardoim et al., 2015). Recent research on the microbial ecology of grapevine plants describes their bacterial microbiota as highly diverse, with specific communities colonizing the different plant parts (Campisano et al., 2014; Zarraonaindia et al., 2015). It should be noted that these bacterial communities can vary according to the particular type of pest management employed (Campisano et al., 2014). Bacteria have been characterized from roots, stems and leaves, as well as from flowers, fruits, and seeds (Compant et al., 2011; Martins et al., 2013). These studies show that most of the bacterial inhabitants of grapevine plants could proceed from the root systems to the aerial parts, but that some others derive from other parts of the phytosphere before colonizing internally plant tissues (Compant et al., 2011; Hardoim et al., 2015).

In a preliminary study, Bruez (2013) indicated that diverse microflora, including fungi and bacteria, colonized the wood tissues of both asymptomatic and esca-diseased grapevine. This was not surprising, as grapevine hosts endophytic microbes in all plant parts as described before. So far, however, an in-depth knowledge of such bacterial microbiota has been lacking. There is a need to know which kinds of bacteria inhabit the different wood tissues of grapevine plants. There is also no information on differences of bacterial communities inside these tissues, but this should be studied in order to understand better the microflora inhabiting wood tissues. Some bacterial colonizers may have putative role(s) in the esca etiology, and some of them might (i) reduce the esca necroses or (ii) help the pathogenic-GTDs fungi. Both these hypotheses need to be evaluated and studied.

Some reports indicate that in many trees, wood-inhabiting bacteria are associated with wood decay (Clausen, 1996) and that they may degrade lignin (Bugg et al., 2011). This may be true also for grapevine plants subjected to esca disease, but this needs to be demonstrated. In fact, several questions can be related to grapevine subjected to esca disease. We do not know the role(s) of bacteria inside the wood tissues of grapevine, nor their community types. Are the communities different according to the various parts, such as cordon and trunk? Are these tissues colonized by potentially pathogenic or biocontrol bacteria? Are the typical necroses associated with esca development also colonized by bacteria and what do these bacteria in the process of wood degradation? Are wood pathogens limited *in planta* by natural antagonists? Evaluation of these hypotheses needs to be carried out to understand better the esca etiology. We now need not only to focus on fungi, but to try to determine the grapevine plant community as a whole in order to understand, at least in part, what the role of microbes other than fungi on esca development/evolution in grapevine plants could be.

In order to characterize the specific bacterial communities colonizing both the esca-foliar symptomatic and asymptomatic grapevines, four approaches were used in this study: (i) the genetic structure of both types of bacterial communities was studied using a fingerprinting method, Single Strand Conformation Polymorphism (SSCP); (ii) community-level physiological profiles (CLPP), generated with sole-carbon source-utilization tests from Biolog^TM^, which provided physiological data for the bacterial communities of wood; (iii) bacterial diversities within wood units were studied by isolating and sequencing 16S rRNA genes of strains from necrotic and healthy wood tissues; (iv) inoculation of plant cuttings by different bacterial strains inoculated with one of the GTD-associated fungi, *N. parvum*, to determine the positive, neutral or negative role(s) of bacteria on the development of inner wood necrosis and on external canker.

## Materials and Methods

### Plant Material and Sample Processing

The sampling site of grapevine plants was located at the Chateau Luchey-Halde vineyard in Mérignac (Bordeaux region, France). Experiments were carried out, in July 2012, on 12-year-old Cabernet Sauvignon vines (*Vitis vinifera* L.) planted in a sandy-clay soil with 10114MG as rootstock. Plants that had either previously expressed or not the esca-foliar symptoms were selected and then uprooted. Whereas selected foliar-symptomatic plants had previously expressed esca-foliar symptoms at least twice over a period of 4 years (2008–2012), this was not the case for the asymptomatic plants, which had never expressed esca-foliar symptoms.

In order to proceed with downstream analyses, four asymptomatic and four esca-foliar symptomatic plants were collected. For each sampled vine, the cordon and trunk were cut longitudinally to choose the necrotic and non-necrotic wood tissues in function of their presence and localization (**Table 1**). White-rot, a specific necrotic tissue of esca (Maher et al., 2012; Bruez et al., 2014) was only present in the cordon of symptomatic plants (**Table 1**). For all the 29 wood tissues collected, the samples consisted of 2 × 15 wood chips (around 5 mm in length) for microbiological analyses (isolation and Biolog Ecoplates^TM^), and approximately 10 g of tissue for molecular analyses.

**Table 1:** Description of the pathological status of the grapevine wood tissues sampled from asymptomatic and esca-foliar symptomatic plants.

Vine plant	Status	Necrotic tissues in cordon		Non-necrotic tissues		
			
		White-rot	Necrotic tissues	Cordon	Inner trunk	Outer trunk
R26C33	Asymptomatic		X		X	X
R37C18	Asymptomatic		X		X	X
R4C8	Asymptomatic		X	X	X	X
R70C30	Asymptomatic			X	X	X
R43C24	Symptomatic	X		X	X	X
R38C45	Symptomatic	X		X	X	X
R53C29	Symptomatic	X	X		X	X
R67C24	Symptomatic		X	X	X	X


### Analysis by SSCP of the Bacterial Communities Colonizing the Grapevine Wood

All the wood samples were ground in liquid nitrogen with a one-ball mill of Dangoumau type and kept at -80°C prior to DNA extraction. DNA was extracted from 60 mg aliquots of woody tissues using the Indvisorb Spin Plant mini Kit (Invitek) in accordance with the manufacturer’s instructions. The DNA extracts were then quantified with a nanodrop (ND-1000, Thermoscientific, Labtech) and homogenized at a concentration of 10 ng/μl. DNA was extracted in duplicate for each of the 29 wood samples collected.

A pair of primers recognizing the V5–V6 region of the 16S rRNA gene was used, 799f (AACMGGATTAGATACCCKG) and 1115r (6-FAM-AGGGTTGCGCTCGTTG) (Redford et al., 2010). DNA was amplified by PCR in an Epgradient Mastercycler (Eppendorf) in a reaction mixture (25 μl final volume) consisting of 1 μl of DNA template (10 ng/μl), 2.5 μl of 10X Pfu buffer (Agilent Technologies), 1 μl of dNTP (10 mM), 0.5 μl of each primer (20 μM), 2.5 μl of BSA (10 μg/μl) (New England BioLabs), 0.5 μl of Pfu Turbo (Agilent Technologies), and 16.5 μl of sterile distilled water. The cycling conditions were: enzyme activation at 95°C for 2 min; 25 cycles of denaturation at 95°C for 45 s; hybridization at 54°C for 30 s; extension at 72°C for 1 min; a final extension at 72°C for 10 min. The PCR products were visualized using 2% TBE agarose gel electrophoresis prior to SSCP analysis.

Single Strand Conformation Polymorphism analyses were performed on an ABI PRISM 3130 Genetic Analyzer (Applied Biosystems) equipped with four 36-cm long capillaries. One microliter of a PCR product was mixed with 18.8 μl formamide Hi-Di (Applied Biosystems) and 0.2 μl standard internal DNA molecular weight marker Genescan 400 HD ROX (Applied Biosystems). The sample mixture was denatured at 95°C for 5 min and immediately cooled on ice, and then loaded onto the instrument. The non-denaturing polymer consisted of 5.6% POP conformational analysis polymer (Applied Biosystems), 10% glycerol, EDTA buffer 10x (Applied Biosystems), and water. The migration time was set to 2000 s, the voltage to 15 kV, and the temperature was 32°C.

Samples were co-migrated with the fluorescent size standard (GeneScan-400 ROX) to allow comparison of migration profiles between samples. Patterns were aligned with StatFingerprints (version 2.0) and studied using principal components analyses (PCA) (Vallance et al., 2012).

### CLPP Analyses of Bacterial Communities

Bacterial communities were characterized by their metabolic fingerprints, using Biolog Ecoplates^TM^ (AWEL International). This method allows the discrimination of heterotrophic microbial communities of environmental samples by comparing C-utilization profiles through 31 lyophilized substrates. These, together with their individual negative control, were present in triplicate in each plate (Garland and Mills, 1991; Janniche et al., 2012).

Microplates were inoculated with 15 ml suspensions (150 μl/well) prepared as follows: 15 pieces of wood of each sample were incubated for 48 h at 27°C in Tryptic Soy Broth (TSB). After centrifugation at 3000 rpm for 30 min at 15°C, bacterial pellets were re-suspended in 15 ml 0.85% NaCl solution. Plates were then incubated at 27°C in the dark. Optical density (OD) readings were performed at a wavelength of 590 nm after 48 h of incubation (Multimode microplate reader, Synergy HT, Biotek, USA).

### Isolation of Bacteria

Wood fragments were surface-disinfected by immersion in 70% ethanol, followed by 2.5% calcium hypochlorite solution for 1 and 3 min, respectively, for most of the samples, or for 2 × 20 s for the white-rot ones. Samples were then rinsed three times with sterile distilled water, dried on sterile filter paper and 15 sterilized chips (3 × 5 chips) were plated onto R2A agar amended with cycloheximide (Sigma) for 48 h at 27°C. Two hundred seventeen bacterial strains were isolated and subsequently purified onto new R2A agar plates before being maintained on cryogenic storage beads (Cryosystem Protect, Dutscher) at -20°C. In order to check the surface sterilization, 3×100 μl of the final rinse water were spread on R2A agar plates and also incubated for 48 h at 27°C.

### Identification of Bacteria by Sequencing the 16S rRNA Gene

The DNA of the bacterial strains was extracted with a CTAB chloroform/isoamyl alcohol (24:1) protocol using 48-h-old TSB cultures. DNA extracts were then quantified with a nanodrop (ND-1000, Thermoscientific, Labtech) and homogenized at a concentration of 50 ng/μl.

The DNA was amplified by PCR in a Mastercycler Gradient Thermocycler (Eppendorf) in a reaction mixture (30 μl final volume) consisting of 2 μl of DNA template (10 ng/μl), 3 μl of 10X reaction buffer (Eurogentec), 1 μl of MgCl_2_ (50 mM), 0.6 μl of dNTP (10 mM), 0.6 μl of each primer (10 μM), i.e., 799f (AACMGGATTAGATACCCKG) and 1492r (GTTACCTTGTTACGACTT), 3 μl of BSA (10 μg/μl) (New England BioLabs), 0.1 μl of SilverStar DNA polymerase (Eurogentec) and 19.1 μl of sterile distilled water. The cycling conditions were: enzyme activation at 94°C for 3 min; 30 cycles of denaturation at 94°C for 1 min; hybridization at 50°C for 45 s; extension at 72°C for 1 min; a final extension at 72°C for 8 min. The amplicons were sent to GATC Biotech (Germany) to sequence the 16S rRNA gene with the 799f and 1492r primers. For species level identification, sequences were compared with the Genbank database by using the Blastn program (Altschul et al., 1997), with a 99% similarity cutoff. Sequences were deposited in the NCBI databank under the accession numbers KT247611 to KT247643.

### *In Vitro* and *in Vivo* Pathogenicity Tests of Selected Bacterial Strains in the Presence of *Neofusicoccum parvum*

For these tests, a highly virulent isolate of *N. parvum* “Cou 02” was selected from the collection of INRA-UMR SAVE (Bordeaux) because this species has been considered as one of the most pathogenic among the fungal species associated with GTDs (Urbez-Torres et al., 2009). It was stored at 4°C on Malt Agar (MA) medium and then sub-cultured, 6–7 days before artificial inoculation, on MA Petri dish plates and incubated at 22°C (12 h light/12 h dark).

Fourteen potentially protective or pathogenic bacteria, from the 217 bacterial strains, were chosen according to their status on grapevines and other plants.

### *In Vitro* Test

Dual cultures were used to test the effect of the 14 bacterial strains on fungal growth of *N. parvum*. A loop of bacterial cells of 1-day-old cultures grown on TSA agar dishes was streaked on a 9 cm Petri dish containing potato dextrose agar (PDA) medium. Sterile inoculation loops were used to transfer the tested strain by placing it, as a line, at approximately 2.5 cm from the dish center on one side only of the Petri dish. Then, 24 h later, one mycelial plug of *N. parvum* (4 mm of diameter) was inoculated at the center of each Petri dish. As control, a set of dishes was inoculated similarly, but with the pathogen only. Three replicate dishes per bacterial strain with the pathogen combination were set up. The dishes were then incubated at 23°C. The radial mycelial growth of the pathogen (measured in millimeters) was assessed after 4 days, and the inhibition percentage was then calculated using the following growth inhibition equation (GI%): GI% = 100^∗^(R2 – R1)/R2.

“R1” represents the minimal distance between the mycelial plug center and the fungal colony margin in the direction of the antagonistic bacteria. The control value for fungal colony radius was “R2” assessed, in the same Petri dish, as the distance between the mycelial plug center and the fungal colony margin on the opposite side of the bacteria. Bacterial strains were considered as ineffective in suppressing the pathogen when *R*1 > 2 cm, i.e., without any noticeable inhibition zone.

### *In Vivo* Test

A total of 256 cuttings of cv. Cabernet Sauvignon were processed as previously described (Laveau et al., 2009). The plants were grown for 97 days in an open greenhouse before assessment. Three conditions were applied: (i) control, named uninoculated control (inoculated with a sterile MA plug); (ii) cuttings inoculated with the fungus, *N. parvum* (inoculated control); (iii) cuttings inoculated with 40 μl of sterile bacterial culture medium of the 14 bacterial strains and a plug of fungus. The trial layout was a completely randomized block design with four blocks, each containing 64 plants. Four plants per block were used for each condition.

After an incubation period of 99 ± 5 days, the presence (incidence) of the external canker was assessed visually on each cutting. The bark was then removed, and the stem of each plant was cut longitudinally (from upper part to basal part). The length of the internal vascular lesions or necroses in the cutting was recorded by measuring the necrotic lesions, upward and downward, from the wound-inoculation hole. The length of necrosis and the incidence of external cankers were compared between the control and the different bacteria by using an ANOVA test followed by a Newman–Keuls *post hoc* test in Statbox software.

### Statistical Analyses

#### SSCP Analyses

Patterns were aligned with StatFingerprints (version 2.0) and were gathered in a single numerical database before being statistically described by a global PCA with R (version 2.14.2). Each sampling point was calculated by analyzing 250 variables corresponding to the SSCP profile scans of each sample. PCA was performed using the correlation coefficient of Pearson. Variables with a cos^2^ ≥ 0.5 on one of the first two principal components (Dim1 and Dim2) were estimated as sufficiently well represented by the principal plan generated by this PCA.

A discriminant analysis was performed to compare results for the necrotic tissues of symptomatic and asymptomatic plants using R Software for a MANOVA.

#### CLPP Analysis

After reading the OD at 590 nm, to minimize the effect of differences in density between plates, data were standardized as follows: the average well color development (AWCD) was calculated for each plate and then the blanked absorbance value of each well was divided by the AWCD of the corresponding plate to get a corrected OD value (Garland and Mills, 1991). All corrected OD values were set to fall within 0 and 2 (boundary limits), and were then used for PCA analyses with R (version 3.1.3.). Thirty-one variables per sample were used for the PCA.

A discriminant analysis was performed to compare the samples of necrotic and non-necrotic tissue of cordon from symptomatic plants.

#### Alpha-diversity

Species diversity was calculated using various indexes: the Shannon diversity index, H′ (Shannon and Weaver, 1963; Buckland et al., 2005) and the Simpson diversity index, D. These indexes were calculated using the package Agricolaea of R (version 2.14.2).

## Results

### Status of the Wood of Esca-foliar Symptomatic and Asymptomatic Grapevines

**FIGURE 1:**
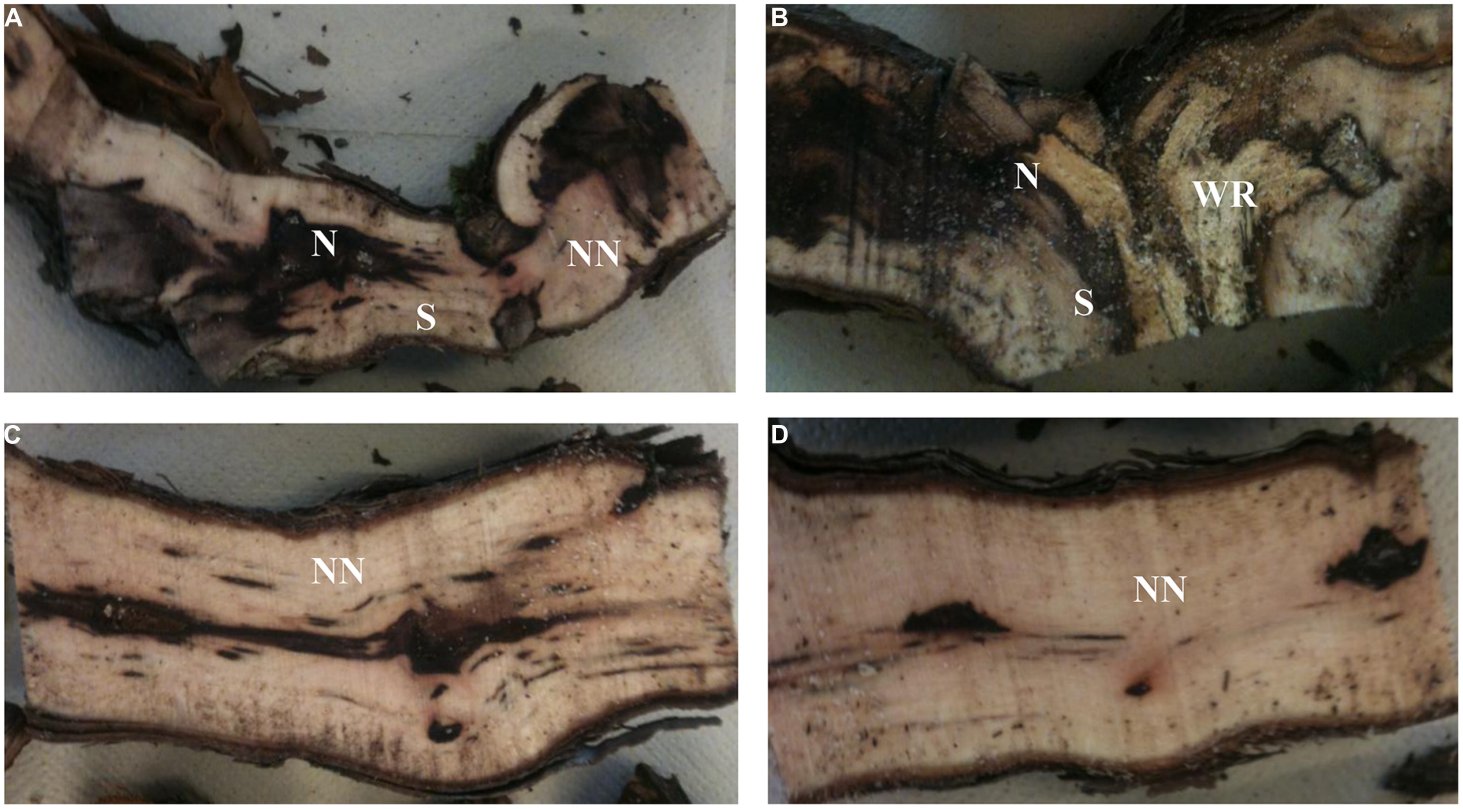
**Longitudinal sections of cordons **(A,B)** and trunks **(C,D)** of plants that had expressed **(B,D)** or not esca-foliar symptoms **(A,C)**.** N, Necrotic tissue; S, Stripes; NN, Non-Necrotic tissue; WR, White-rot.

Examination of longitudinal sections of vines showed that the cordon of the esca-foliar symptomatic plants seemed to have more necroses than the cordon of asymptomatic ones. Non-necrotic tissues were predominant in the trunk of both types of plants. A specific necrotic tissue, white-rot, was only observed in cordon of grapevines that had previously expressed esca-foliar symptoms (**Table 1**). Small dark necrosis was located in the center of the trunk, for both symptomatic and asymptomatic plants (**Figure 1**).

### SSCP Analyses of the Bacterial Communities Colonizing the Grapevine Wood Tissues

The SSCP profiles of the 29 samples that represent the combination of different vine status, anatomical part and tissue status revealed a complex of bacterial communities (data not shown) based on the number of peaks and the relative height of the baseline.

**FIGURE 2:**
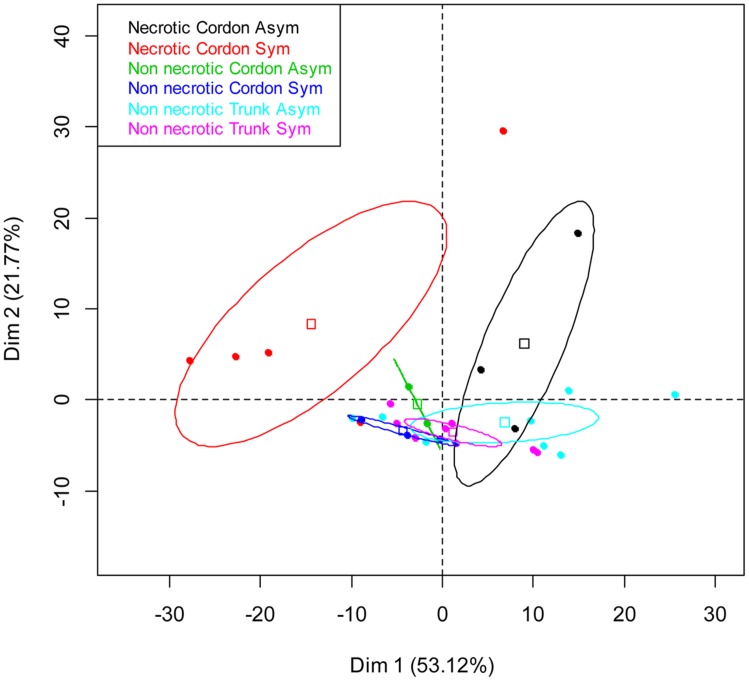
**Principal component analysis (PCA) of the bacterial communities colonizing the non-necrotic and necrotic tissues of trunk and cordon of esca-foliar symptomatic and asymptomatic vines based on SSCP profiles.** The variation (%) explained by each PCA axis is given in brackets. PCA eigenvalues indicate that the first two components, Dim 1 and Dim 2, account for 75% of the total bacterial variability. Squares correspond to individual SSCPs and ellipses to the 95% confidence intervals calculated for each community.

A PCA was carried out to compare the genetic structure of the bacterial communities in terms of tissue types, their localization and vine status. The distribution of samples on the principal plan generated by the PCA analysis is represented in **Figure 2.** Differences in the genetic structure of the bacterial communities were observed in the non-necrotic and necrotic tissues of the cordon for both symptomatic and asymptomatic plants. The different clusters are separated by the first PCA axis, Dim 1, which represents 53% of total bacterial variability, and the ellipses do not overlapped. Furthermore, bacterial communities colonizing the necrotic tissues of cordon from symptomatic and asymptomatic vines differ according to the Dim 1. No distinctive patterns were observed for the non-necrotic tissues of trunk.

### CLPP Analyses of the Bacterial Communities

**FIGURE 3:**
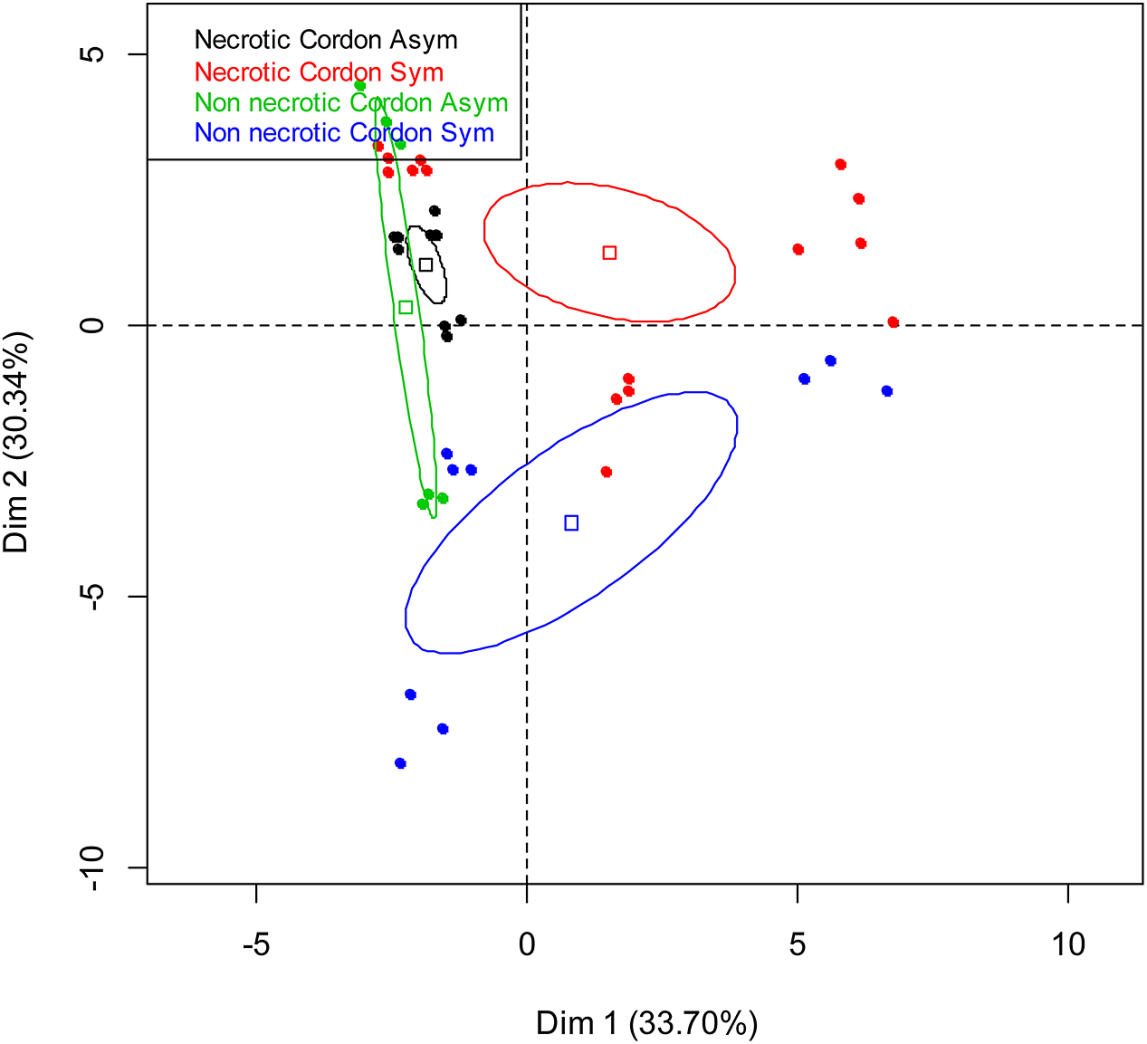
**Principal component analysis of the bacterial communities colonizing the non-necrotic and necrotic tissues of cordons of esca-foliar symptomatic and asymptomatic vines based on Biolog Ecoplates^TM^ AWCD values.** The variation (%) explained by each PCA axis is given in brackets. Squares correspond to individual Community-Level Physiological Profiling (CLPPs) and ellipses to the 95% confidence intervals calculated for each community.

Comparison of C-utilization patterns of the 13 samples (**Table 1**) from the cordon of esca-foliar symptomatic and asymptomatic vines was obtained using PCA of Biolog Ecoplates^TM^ data. **Figure 3** shows the distribution of samples on the principal plan generated by the PCA. PCA eigenvalues indicate that the first two components, Dim 1 and Dim 2, explain 64% of the total data variance. Although the bacterial communities colonizing the cordon necrotic and non-necrotic tissues in asymptomatic plants could not be consistently separated through Dim 1, results showed that bacteria in the cordon had different catabolic activities, according to the type of tissue, necrotic or non-necrotic, and the status of the vines, asymptomatic or esca-foliar symptomatic. No distinctive patterns were observed for the trunk samples (data not shown).

### Identification and Distribution of the Bacteria Isolated from the Wood Tissues

Four hundred thirty-five pieces of wood were cultured from the eight vines collected, and two hundred seventeen bacteria were recovered. Ninety-nine bacterial strains were isolated from asymptomatic plants and one hundred eighteen strains from esca-foliar symptomatic ones.

**FIGURE 4:**
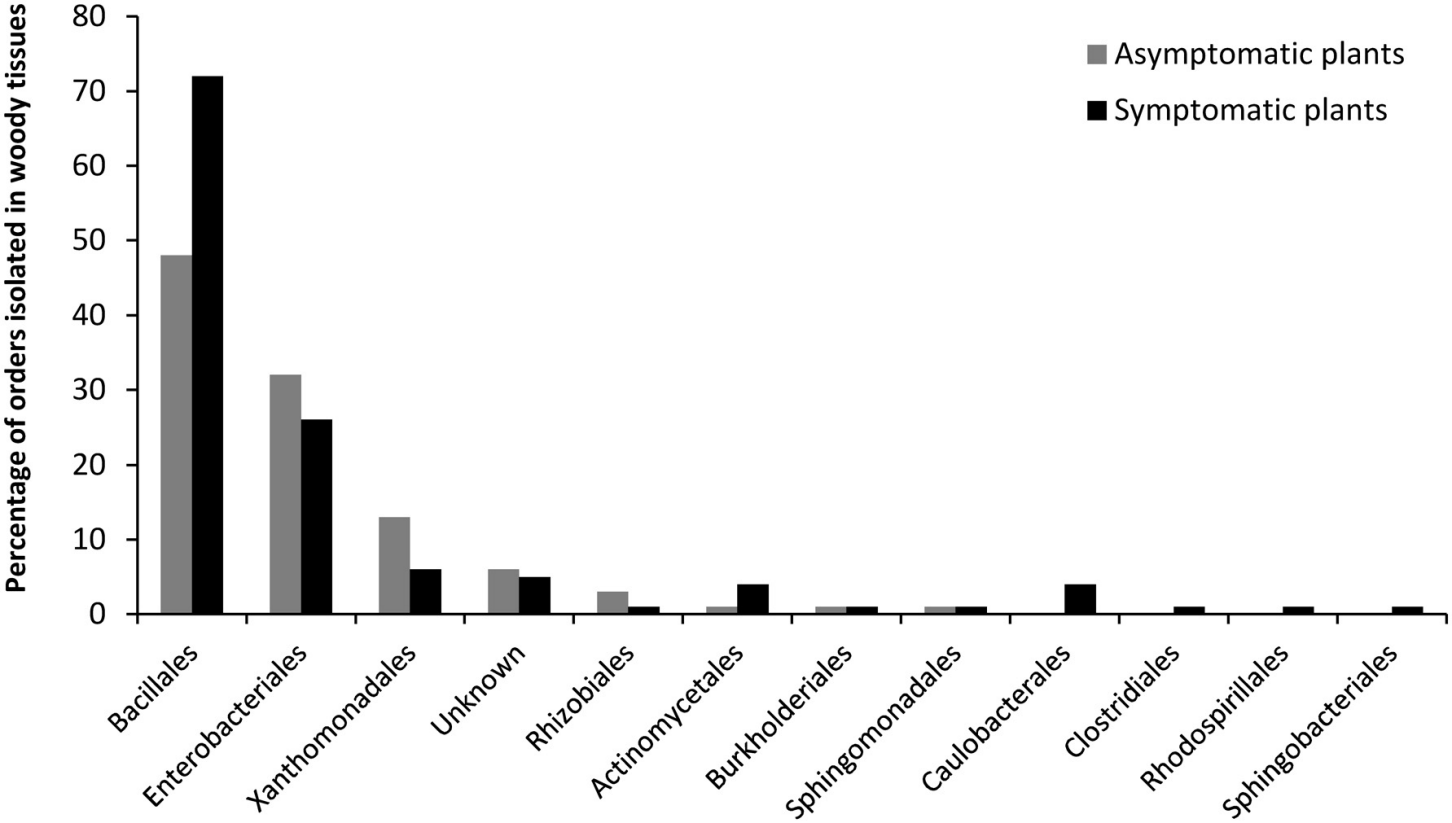
**Identified bacterial orders in the collected vines.** Bacteria isolated from the asymptomatic plants are in gray and those isolated from esca-foliar symptomatic plants are in black.

The most frequently isolated phyla were *Firmicutes* and *Proteobacteria*, mainly comprised of orders such as *Bacillales*, followed by *Enterobacteriales* and *Xanthomonadales*. **Figure 4** shows the distribution of the orders in the eight grapevine plants sampled in July 2012. Eleven orders were described; seven were isolated from the asymptomatic plants and eleven from the esca-foliar symptomatic ones. The most representative orders were the *Bacillales* (60% of the bacterial strains), and 48% of them were isolated from the asymptomatic and 52% from the esca-foliar symptomatic plants. For *Enterobacteriales* (26.7% of the bacterial strains), 55.2% of them were isolated from the asymptomatic plants and 44.8% from the esca-foliar symptomatic plants; for the *Xanthomonadales* (8.8% of the bacterial strains) 74% of them were isolated from the asymptomatic plants and 26% from the esca-foliar symptomatic plants.

**FIGURE 5:**
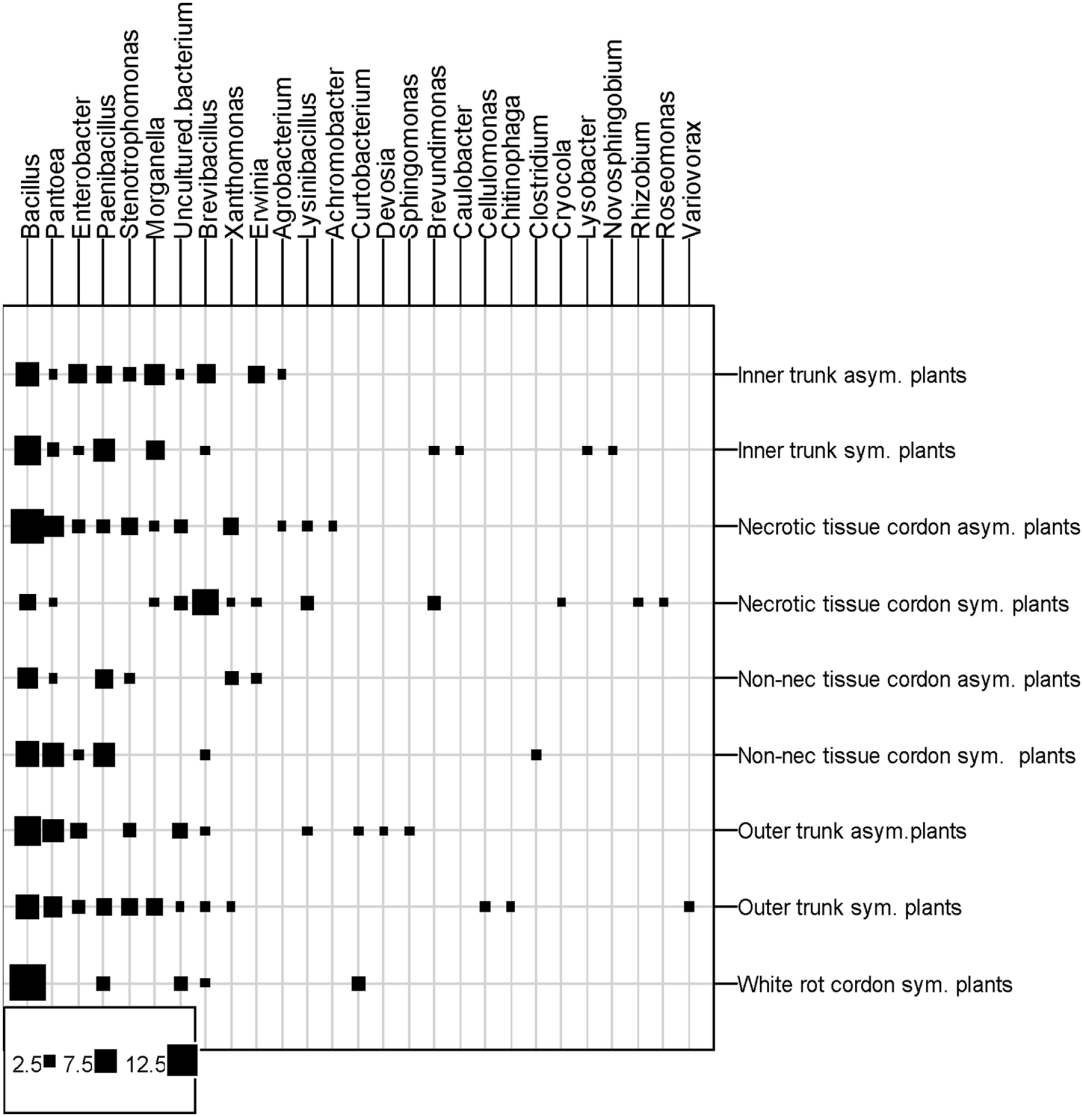
**Distribution of bacterial genera in the different vine parts.** Squares represent the number of isolates per tissue type, plant parts and vine statut. Asym, Asymptomatic plants; Sym, esca-foliar Symptomatic plants; Non-nec, Non-necrotic wood.

Twenty-six genera were identified and isolated from the wood of the eight grapevine plants (**Figure 5**). Fifteen of these genera were isolated from the asymptomatic plants and twenty-three from the esca-foliar symptomatic ones. The nine more numerous genera were isolated in the two types of plants. The most numerous genera were *Bacillus* (34% of the bacterial strains), followed by *Pantoea* (12%)*, Paenibacillus* (9%), and *Enterobacter* (6%). When distribution of the bacterial genera in the wood tissues of plants was considered, *Bacillus* was present in all the samples. The same result was found for *Pantoea*, except that it was not isolated in the white-rot of the cordon. *Paenibacillus* and *Brevibacillus* were not detected in two of the nine wood tissues sampled. Some of the other genera were isolated only in one particular part or type of plant, with a number of isolates generally below five; for example: *Variovorax* and *Cellulomonas* isolated from the trunk of symptomatic vines, *Curtobacterium* and *Sphingomona*s isolated from the asymptomatic vines and *Roseomonas* and *Brevundimonas* in the necrotic tissue of symptomatic vines. The results of the ANOVA test showed that *Enterobacter* sp. (*p* = 0.0346) and *Morganella* sp. (*p* = 0.0339) were more isolated in the trunk than in the cordon.

**Table 2** showed the distribution of the different genera/species according to the wood tissues sampled and the status of the plants. Eighteen of the twenty strains isolated from the white-rot belonged to the *Bacillus* genus, i.e., 6 *Bacillus* sp., 5 *Bacillus ginsengihumi*, 3 *B. licheniformis*, 2 *Paenibacillus* sp., 1 *B. pumilus*, 1 *Brevibacillus reuszeri* (data not shown). These genera were also isolated from both types of wood tissues.

**Table 2:** Bacterial species ranked by the different plant parts and the status of the vines (16S rRNA gene sequencing of the isolated bacteria).

	Asymptomatic plants			Symptomatic plants		
		
Bacterial species	Non- necrotic tissue of trunk	Non- necrotic tissue of cordon	Necrotic tissue of cordon	Non- necrotic tissue of trunk	Non- necrotic tissue of cordon	Necrotic tissue of cordon
*Bacillus* sp. (31)	5	3	5	9	1	8
*Pantoea agglomerans* (26)	7	1	5	6	6	1
*Paenibacillus* sp. (19)	3	4	1	5	4	2
*Brevibacillus reuszeri* (17)	5			2	1	9
*Bacillus licheniformis* (15)	7	1	1	2	1	3
*Morganella morganii* (14)	5		1	7		1
*B. ginsengihumi* (13)		1	1	2	4	5
*Enterobacter* sp. (13)	7		2	3	1	
*B. pumilus* (11)	1		5	3	1	1
*Stenotrophomonas* sp. (11)	4	1	3	3		
*Xantho/Pseudomonas* sp. (6)		1	3	1		1
*Erwinia billingiae* (5)	3	1				1
*P. lautus* (3)				3		
*Agrobacterium* (2)	1		1			
*B. aminovorans* (2)	2					
*Brevundimonas* sp. (2)						2
*Curtobacterium* sp. (2)	1					1
*Lysinibacillus* sp. (2)	1		1			
*Lysinibacillus sphaericus* (2)						2
*P. turicensis* (2)					2	
*Achromobacter* sp. (1)			1			
*B. firmus* (1)						1
*Brevundimonas* sp. (1)				1		
*Caulobacter* sp. (1)				1		
*Cellulomonas* sp. (1)				1		
*Chitinophaga* sp. (1)				1		
*Clostridium* sp. (1)					1	
*Cryocola* sp. (1)						1
*Curtobacterium* sp. (1)						1
*Devosia* sp. (1)	1					
*Lysobacter* sp. (1)				1		
*Novosphingobium* sp. (1)				1		
*P. barengoltzii* (1)				1		
*P. polymyxa* (1)			1			
*Rhizobium* sp. (1)						1
*Roseomonas* sp. (1)						1
*Sphingomonas* sp. (1)	1					
*Variovorax* sp. (1)				1		
*Xanthomons translucens* (1)		1				
**Total number of isolates**	**54**	**14**	**31**	**54**	**22**	**42**


Another isolated species, *Pantoea agglomerans* (26 strains, 12% of all the strains), was isolated from the various wood tissues sampled except for the white-rot. Although the number of bacterial strains isolated from esca-foliar symptomatic plants was higher than in asymptomatic grapevines for *Paenibacillus* sp. (8/11 = asymptomatic/symptomatic), *B. reuszeri* (5/12 = asymptomatic/symptomatic) and *Morganella morganii* (6/8 = asymptomatic/symptomatic), it was not statistically different.

Other genera, e.g., *Enterobacter, Stenotrophomonas, Xanthomonas, Erwinia*, and *Agrobacterium*, were more isolated in the asymptomatic plants than the symptomatic ones, but the number of strains isolated was very low, from 2 to 8. Three strains belonging to *Cellulomonas* sp., *Clostridium* sp., and *Rhizobium* sp. were only detected in esca-foliar symptomatic plants.

### Alpha-Diversity of the Bacterial Genera

**FIGURE 6:**
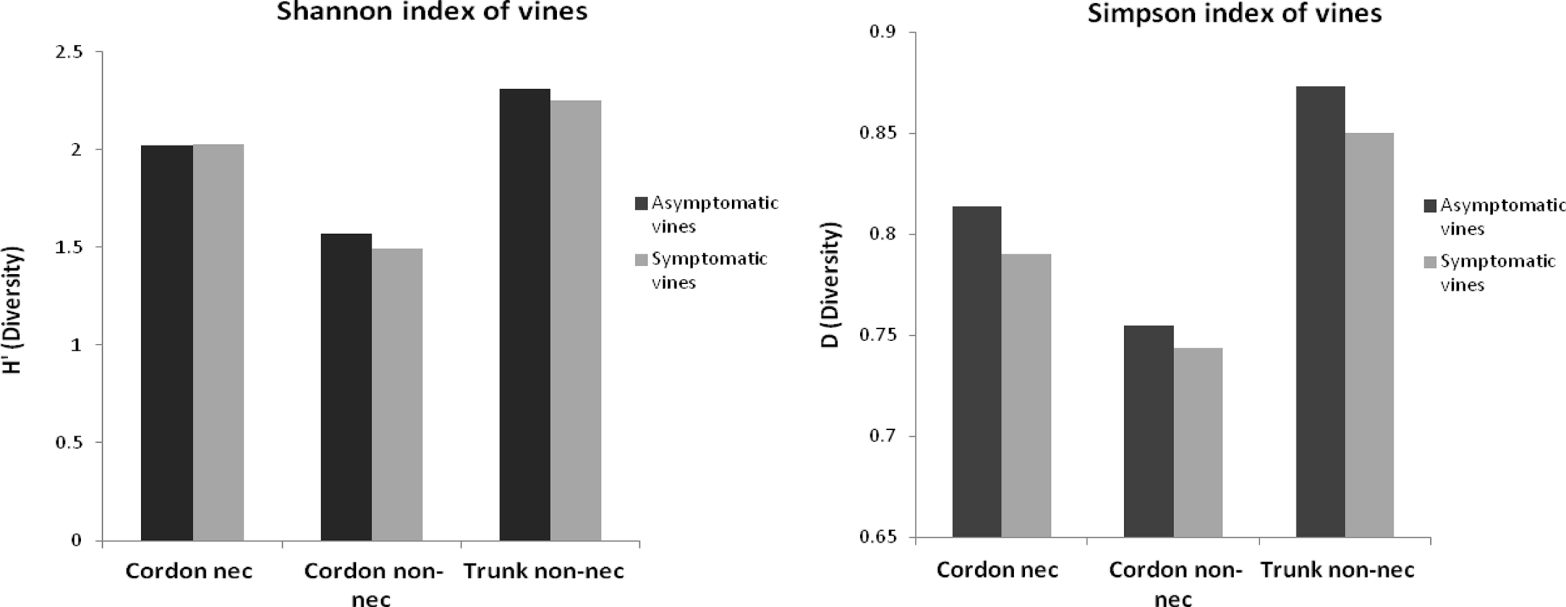
**Alpha-diversity of the bacterial genera isolated from the woody tissues of esca-foliar symptomatic (in gray) and asymptomatic vines (in black)**.

In order to calculate the biodiversity index (**Figure 6**), the number of species was adjusted for the total number of samples. The Shannon indexes showed that the number of bacterial species isolated from all samples is important because the number is higher than 1.49. Depending on the part of the plant, bacterial biodiversity in the non-necrotic tissue of trunk (H′ symptom = 2.25 and H′ asymptom = 2.31) was higher than in the necrotic tissue (H′ = 2 for esca-foliar symptomatic and asymptomatic plants) and non-necrotic tissue of cordon (H′ symptom = 1,49 and H′ asymptom = 1.57). The Simpson indexes, close to 1, showed that for each species isolated, an efficient number of strains were isolated in all samples; the indexes were *D* > 0.74.

### Development of Necrosis by a Fungus, *N. parvum*, Inoculated with Bacteria Isolated from Wood Tissues

**FIGURE 7:**
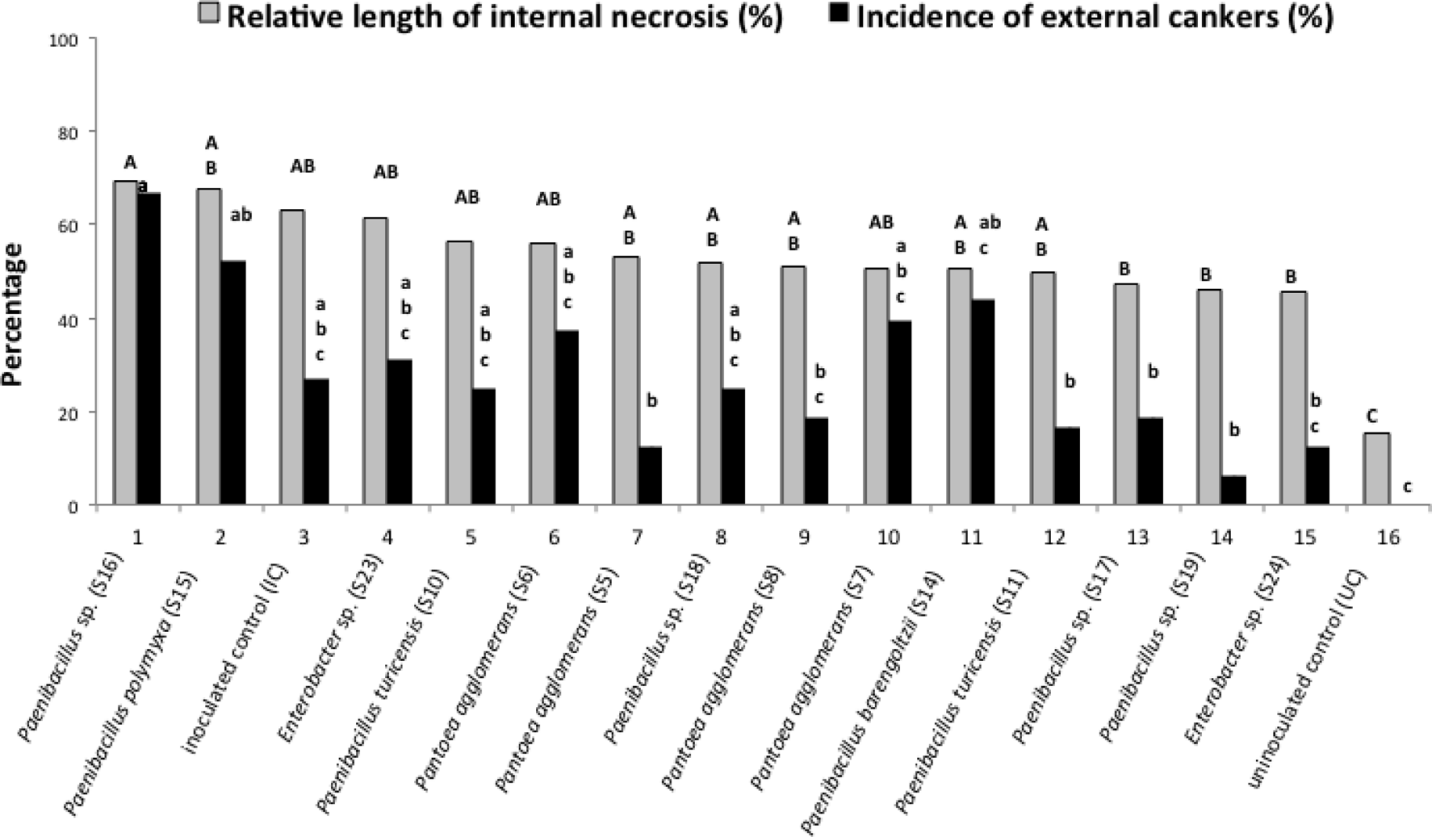
**Effect of different bacterial isolates on the development of wood inner necrosis and external canker on cuttings with inoculated the fungus *Neofusicoccum parvum*.** In gray, the results represent the percentage of the length of internal necrosis for cuttings inoculated or not with the fungus and one of the bacteria. The external cankers (under the bark), are represented in black. Different letters (capital letters for gray results and small letters for black results) indicate significant differences (*P* < 0.05) in accordance with the ANOVA and Newman–Keuls tests.

**Figure 7** shows the development of the necroses in the presence of *N. parvum* and bacteria. All the tested bacteria inoculated with the fungus did not increase or decrease significantly external and internal cankers in comparison with the cuttings inoculated only by the fungus. The *in vitro* pathogenicity assay showed, however, that four bacterial strains, S11 (*Paenibacillus turicensis*), S15 (*P. polymyxa*), S16 (*Paenibacillus* sp.), and S19 (*Paenibacillus* sp.), did have an effect on fungus development. For these four strains, the GI (Growth inhibition percentage) was at least 25%. Strains S15, S16, and S19 had a growth inhibition percentage of 25% and, strain S11 had 32% percent inhibition. The ANOVA test showed that there was no difference in growth inhibition between the four strains even if the strain S11 had a better growth inhibition.

## Discussion

Comparison of bacterial communities from esca-foliar symptomatic and asymptomatic grapevines was performed for the first time. Bacterial communities of two anatomical parts (trunk and cordon) and tissue status (necrotic and non-necrotic) were analyzed for both symptomatic and asymptomatic plants using four different approaches, the SSCP, the CLPP, the isolation and sequencing of strains and the experimentation to determine the role of some bacteria.

Cutting the plants longitudinally allowed us to observe and choose the right tissues to show that, in the wood tissues of the young grapevines (12-year-old) sampled (i) there were no sign of necrotic tissues, except for a small central necrosis in the trunk and (ii) white-rot, a typical necrotic tissue, was observed only in the cordon of symptomatic plants.

Maher et al. (2012) showed that for mature (20-year-old) or old (30-year-old) vines, central necrosis was present in the center of the trunk. Their logistic model confirmed that white-rot in cordon is the best predictor for the chronic form of esca. Our finding is also in agreement with the early descriptions of this disease, which frequently mentioned the transformation of healthy wood into white-rot (Larignon and Dubos, 1997; Bruez et al., 2014).

In the necrotic and non-necrotic tissues of the cordon and the non-necrotic tissues of the trunk, we observed a complex of bacterial microflora. The fingerprinting method, SSCP, showed that, depending on the wood samples, the bacterial communities were different for necrotic and non-necrotic tissues. Different bacterial communities for various tissue types, commonly found for fungal microflora (Maher et al., 2012), were reported here, for the first time.

Biolog Ecoplates^TM^ were used to study the metabolism of the bacteria, as regards both tissue and plant types. The results for the bacteria colonizing the cordons of foliar-esca symptomatic plants showed that the bacterial metabolism was tissue-dependent. The bacteria of white-rot, necrotic and non-necrotic tissues metabolized differently the carbon substrates of the Biolog Ecoplates^TM^, thus indicating that they were adapted according to the tissue types. Fritze et al. (2000) showed that the trophic behaviors of the bacterial communities using the carbon substrates were different according to the type of humus. Biolog Ecoplates^TM^ also allowed us to differentiate bacterial communities from the non-necrotic outer and inner wood tissues of the trunk.

From all the wood tissue samples, the most abundant genus isolated was *Bacillus*. Of the nine most abundant species, six belonged to the *Bacillus* genus, i.e., *Bacillus* sp. (14% of the total of strains), *Paenibacillus* sp. (12%), *B. reuszeri* (8%), *B. licheniformis* (7%), *B. ginsengihumi* (6%), and *B. pumilus* (5%). Recent studies have reported that this genus seems to be a frequent and common colonizer of various grapevine organs. West et al. (2010) showed that *Bacillus* was the most abundant genus in the cultivar Chardonnay. Compant et al. (2011) also isolated several members of this genus in the reproductive organs and the grapevine roots at two different vegetation stages. Strains of *Bacillus* have great potential for biological control, and their effectiveness in controlling multiple plant diseases has been reported by many scientists (Gueldner et al., 1988; Paul et al., 1998; Emmert and Handelsman, 1999; Collins et al., 2003; Toure et al., 2004). Among the most abundant *Bacillus* species we identified, *B. licheniformis* has been used to control pathogens on many plants (Lee et al., 2006).

Two roles should be assigned to the bacteria isolated from the necrotic and non-necrotic tissues of wood: (i) a positive role, due to the biocontrol potential that many species have; (ii) a negative one, by predisposing the vine wood to fungal attacks. The present experiment, in which bacterial strains were co-inoculated with *N. parvum* as a pathogen in the wood of vine cuttings does not, however, show a positive or a negative role of bacteria on canker development.

The second most frequent bacterial species isolated in our experiment was *P. agglomerans*, a Gram-negative bacterium, initially defined as epiphyte (Lindow and Brandl, 2003), but also able to colonize the roots (Francis et al., 2000). We isolated *P. agglomerans* from the necrotic and non-necrotic wood tissues of both cordon and trunk. Compant et al. (2011) isolated the bacteria from the endorhiza at the flowering stage of the plant, and Bell et al. (1995) found that 19% of all the bacteria collected from the xylem were *P. agglomerans*. Like the *Bacillus* species mentioned above, strains of *P. agglomerans* display biocontrol activity on various plants (Francis et al., 2000), including the control of *Botrytis cinerea* infections on vine (Magnin-Robert et al., 2007; Trotel-Aziz et al., 2008; Verhagen et al., 2011). Due to the production of metabolites responsible for their biocontrol activities, *Bacillus* sp. and *P. agglomerans* could interact in the grapevine wood tissues with the other microorganisms, particularly the numerous fungal species, plant pathogenic or not. Thus, depending on the bacterial communities and the fungi they interact with, development of esca/BDA may be delayed, prevented or not. However, this is still speculation, as inoculation of bacteria together with *N. parvum* does not allow *in planta* biocontrol activities to be seen. Only a small set of microbes has been, however, used in this study, and further research should be carried out with a larger set of bacteria to see if some of them can reduce GTD development, but also to determine the negative role of some bacteria on canker development. Could bacteria be involved in the degradation of the wood and in the formation of typical GTD necroses? The bacteria isolated from the woody tissue of grapevine could interact with fungi, particularly those involved in esca/BDA (Bruez, 2013), which are already present in those tissues. Bacteria could affect wood permeability, attack wood structure, or work synergistically with other bacteria and soft-rot fungi to predispose wood to fungal attack (Clausen, 1996). This hypothesis could be envisaged, using the *Sphingomonas* sp*., Erwinia* sp., and *P. polymyxa*, bacteria that we isolated, particularly because they are known to degrade lignin. We also isolated two strains of *Curtobacterium* sp. which, as Kamei et al. (2012) showed, could have a role in the expression of the physiological function of a fungus, *Stereum* sp., involved in GTDs. As the relationships of these bacteria with other GTD-fungi have not been investigated, this could open up other hypothesis of research.

## Conclusion

Our study shows that bacterial communities colonize the grapevine wood of both esca-foliar symptomatic or asymptomatic plants. Certain bacterial species do not induce changes in the development of wood degradation, according to the type of relationship they establish with the fungal microflora, but this merits further research. Determining the succession of microflora would also be helpful in understanding how wood necroses develop within the grapevine.

## Conflict of Interest Statement

The authors declare that the research was conducted in the absence of any commercial or financial relationships that could be construed as a potential conflict of interest.
